# Flexible Polydimethylsiloxane Composite with Multi-Scale Conductive Network for Ultra-Strong Electromagnetic Interference Protection

**DOI:** 10.1007/s40820-022-00990-7

**Published:** 2022-12-29

**Authors:** Jie Li, He Sun, Shuang-Qin Yi, Kang-Kang Zou, Dan Zhang, Gan-Ji Zhong, Ding-Xiang Yan, Zhong-Ming Li

**Affiliations:** 1 College of Polymer Science and Engineering, State Key Laboratory of Polymer Materials Engineering, Sichuan University, Chengdu, 610065 People’s Republic of China; 2 School of Aeronautics and Astronautics, Sichuan University, Chengdu, 610065 People’s Republic of China

**Keywords:** Flexible conductive polymer composites, Silver-plated polylactide short fiber, Carbon nanotube, Electromagnetic interference shielding, Multi-scale conductive network

## Abstract

**Highlights:**

- A multi-scale conductive network was constructed in flexible PDMS/Ag@PLASF/CNT composite with micro-size Ag@PLASF and nano-size CNT.
- The PDMS/Ag@PLASF/CNT composite showed outstanding electrical conductivity of 440 S m^-1^ and superior electromagnetic interference shielding effectiveness of up to 113 dB.
- The PDMS/Ag@PLASF/CNT composites owned good retention (> 90%) of electromagnetic interference shielding performance even after subjected to a simulated aging strategy or 10,000 bending-releasing cycles.

**Abstract:**

Highly conductive polymer composites (CPCs) with excellent mechanical flexibility are ideal materials for designing excellent electromagnetic interference (EMI) shielding materials, which can be used for the electromagnetic interference protection of flexible electronic devices. It is extremely urgent to fabricate ultra-strong EMI shielding CPCs with efficient conductive networks. In this paper, a novel silver-plated polylactide short fiber (Ag@PLASF, AAF) was fabricated and was integrated with carbon nanotubes (CNT) to construct a multi-scale conductive network in polydimethylsiloxane (PDMS) matrix. The multi-scale conductive network endowed the flexible PDMS/AAF/CNT composite with excellent electrical conductivity of 440 S m^−1^ and ultra-strong EMI shielding effectiveness (EMI SE) of up to 113 dB, containing only 5.0 vol% of AAF and 3.0 vol% of CNT (11.1wt% conductive filler content). Due to its excellent flexibility, the composite still showed 94% and 90% retention rates of EMI SE even after subjected to a simulated aging strategy (60 °C for 7 days) and 10,000 bending-releasing cycles. This strategy provides an important guidance for designing excellent EMI shielding materials to protect the workspace, environment and sensitive circuits against radiation for flexible electronic devices.
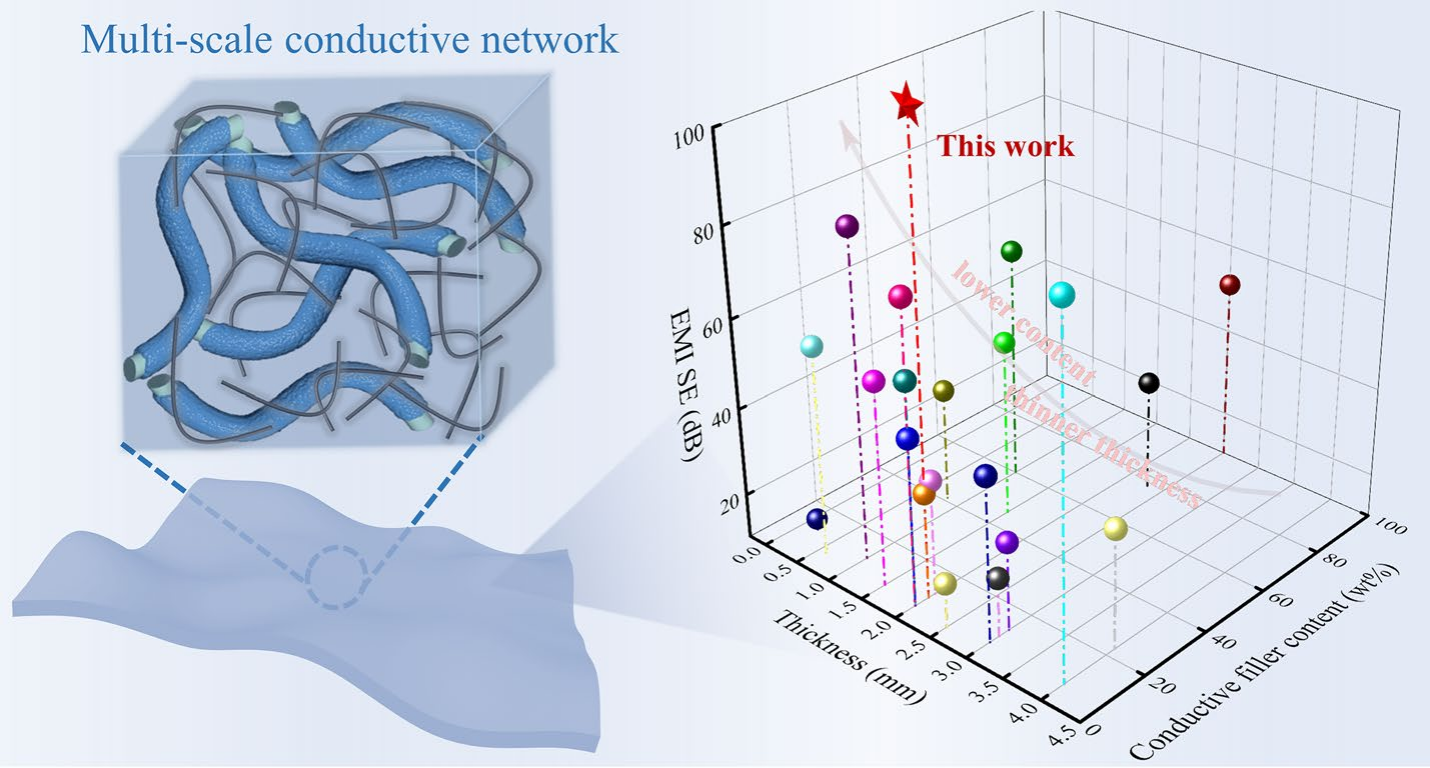

**Supplementary Information:**

The online version contains supplementary material available at 10.1007/s40820-022-00990-7.

## Introduction

With the ferocious development of flexible wireless electronics and widespread use of electromagnetic waves, flexible electromagnetic interference (EMI) shielding materials with ultra-strong electromagnetic interference shielding effectiveness (EMI SE > 90 dB) have attracted more and more attention [1–3, 59–62]. Conductive polymer composites (CPCs) have shown a bright future in the EMI shielding field of smart electronic devices because of their good processability and low density [4–6, 63–66].

Generally, the EMI shielding performance of CPCs is related to the type and content of conductive fillers [7–10, 67–70], which includes metal-based fillers (e.g., silver, copper and nickel nanoparticles or nanowires) [11–13, 71], carbon-based fillers (e.g., carbon black, carbon nanotube and graphene) [14–16], intrinsically conductive polymers (e.g., poly(3,4-ethylenedioxythiophene), polyaniline and polypyrrole) [17–19, 72, 73] and other hybrid fillers [20–22, 42, 74–77]. Carbon nanotubes (CNT), which possess excellent conductivity and high aspect ratio, can be used to construct 3D conductive networks and design EMI shielding composite materials [23, 24]. According to the classical percolation theory and Schelkunoff theory, the primary strategy of improving electrical conductivity and EMI SE of flexible polymer matrix is to increase the CNT content and construct efficient conductive networks [25–27]. Based on this idea, Zeng et al. [28] fabricated a series of flexible waterborne polyurethane/CNT composites with CNT content varying from 16.7 to 80 wt%, and the corresponding EMI SE enhanced from 15 to 80 dB. However, because the flexibility of CPCs usually reduces notably with the increase of CNT content, it is difficult to realize flexible CPCs with ultra-strong EMI SE by uniformly filling high content of CNT into flexible polymers [29–31]. It is also not feasible to achieve the ultra-high EMI shielding performance of CPCs by improving the electrical conductivity of CNT. For instance, Zhang et al. [32] designed CNT decorated with silver (Ag) nanoparticles, and the EMI SE of the composites reached 56 dB, which was still far from meeting the high requirements for the materials with ultra-strong EMI shielding properties.

Constructing an innovative conductive network of flexible CNT-based CPCs with high electrical conductivity seems to be a facile strategy to obtain ultra-strong EMI SE and outstanding flexibility [33–37]. Wang et al. [38] introduced flexible and electrically insulated fibers to construct a conductive network by means of the volume exclusion effect, and the electrical conductivity reached 70 S m^−1^ and the EMI SE increased to 41 dB, while showing satisfactory flexibility. Based on this concept, Liao et al. [39] reported a conductive Ag@GF material and a kind of heterogeneous silicone rubber/(Ag@GF)/ CNT composite foams with a gradient structure, and its electrical conductivity reached 2,809 S m^−1^. The EMI SE reached 78.6 dB and exhibited excellent recoverability, but the complex layer-by-layer combination process limited its wide application. Subject to the design on the CNT-based conductive network of CPCs, it is still not enough to meet the high-level frontier requirements of ultra-strong and flexible EMI shielding composites.

Herein, we constructed a multi-scale conductive network with an Ag-plated polylactide short microfiber (Ag@PLASF, AAF) as a conductive micro-size filler and CNT as a nano-size filler and then filled it evenly into polydimethylsiloxane (PDMS) to prepare flexible CPCs with ultra-strong EMI protection. The average EMI SE of the resultant composites with only 5.0 vol% of AAF and 3.0 vol% of CNT reached as high as 113 dB, far greater than 36 and 43 dB for the composites with single-filled AAF or CNT. Furthermore, the composite material possessed a good retention (> 90%) in EMI shielding performance due to its outstanding flexibility, regardless of being compressed in a simulated aging environment or subjected to bending-releasing cycles of as high as 10,000. This strategy provides an easy-to-process method, that is, constructing a multi-scale conductive network to design flexible CPCs with excellent shielding performance, so as to protect the workspace, environment and sensitive circuits against radiation for flexible wireless electronic devices.

## Experimental Section

### Materials

Polydimethylsiloxane (PDMS, sylgard 184 silicone elastomer, UK) was purchased from the Dow Chemical Company, and its volume resistivity was about 2.9 × 10^14^ Ω cm. Polylactide short microfiber (PLASF, 1.7–771 NBT, Germany) was provided by Advansa GmbH with a linear density of 1.55–1.81 dtex. The average length and diameter of PLASF were 5 mm and 10 μm, respectively. Carbon nanotube (CNT, NC 7000, Belgium) with 90% of carbon purity was provided from the Nanocyl S. A. It had a density of 1.75 g cm^−3^, an average length of 1.5 μm and a diameter of 9.5 nm. The chemical reagents for electroless silver plating were obtained from Chengdu Kelong Chemical Reagent Factory (China), containing silver nitrate (AgNO_3_, AR), glucose (C_6_H_12_O_6_, AR) and stannous chloride (SnCl_2_, AR).

### Preparation of AAF and PAAC Composites

#### Preparation of AAF

PLASF was treated in a mixed solution of methanol/deionized water (v/v, 2/1) containing 1 g L^−1^ of NaOH and stirred magnetically for 48 h. Subsequently, the treated PLASF was passed through suction filtration, washed several times with deionized water and then dried in a vacuum oven. 0.5 g of the treated PLASF was further dispersed in the aqueous solution of SnCl_2_ and stirred for 20 min, and then put into silver ammonia solution (containing 10 g L^−1^ of AgNO_3_). After stirring for 3 min, glucose solution (20 g L^−1^) was added into the mixed solution. After 1 h of continuous chemical reaction, the Ag@PLASF (AAF) was obtained, which was washed several times with deionized water, and dried in a vacuum oven at 80 °C.

#### Preparation of PAAC Composites

Firstly, the PDMS precursor was dissolved in n-heptane, and then the quantitative amount of CNT (1.0, 2.0, 3.0 vol%) were dispersed in the mixed solution by ultrasonic to obtain a CNT suspension. Secondly, AAF in proportion (1.0, 3.0, 5.0 vol%) were added to the above suspension and stirred at 500 rpm for 10 min. Afterward, all materials were dispersed in a beaker with n-heptane and put it into a vacuum oven at 80 °C to completely remove the n-heptane. Finally, the flexible PDMS/AAF/CNT composites were fabricated by hot pressing mixture at 120 °C for 1 h in a mold with the cross-linking agent. For convenience, the PDMS/AAF/CNT composites were labeled as PAA*x*C*y* composites, where *x* and *y* were defined as the volume contents of AAF and CNT in the composites.

### Characterization

The morphology of the AAF and PAAC composites was characterized using a field emission scanning electron microscope (FESEM, Nova Nano SEM450, FEI, USA). The X-ray diffraction curves were obtained on an X-ray diffractometer (XRD, Rigaku Ultima IV, Japan) by using 2 Theta/Theta continuous scanning mode. A thermogravimetric analyzer (TGA, TG209, NETZSCH, Germany) was used to measure the mass variation of PLASF and AAF at 30–800 °C at a heating rate of 10 °C min^−1^ in a nitrogen atmosphere (50 mL min^−1^). The distribution of AAF and CNT in the composites was studied by a transmission electron microscope (TEM, JEOL JEM 2100F, Japan). A four-point probe instrument (Guangzhou Four Point Probe Technology Co., Ltd., China) was employed to assess the electrical conductivity of PAAC composites. A vector network analyzer (VNA, Agilent N5247A, USA) was used to evaluate the EMI shielding performance at the X-band frequency (8.2–12.4 GHz). The samples (13 mm in diameter) were controlled in a holder, which was connected by a coaxial line to separate the VNA ports. The obtained scanning parameters (*S*_11_ and *S*_21_) were summarized to evaluate the EMI shielding mechanism, including total *SE* (*SE*_T_), reflection *SE* (*SE*_R_) and absorption *SE* (*SE*_A_), as well as absorption coefficient (*A*), reflectivity coefficient (*R*) and transmissivity coefficient (*T*) with the following relationships:

1 $$R={|{S}_{11}|}^{2}$$

2 $$T={|{S}_{21}|}^{2}$$

3 $$A+R+T=1$$

4 $${SE}_{R}=-10lg(1-R)$$

5 $${SE}_{A}=-10lgT/(1-R)$$

6 $${SE}_{T}={SE}_{A}+{SE}_{R}+{SE}_{M}$$

where *SE*_M_ represents multiple internal reflections of the microwaves, which can be ignored when *SE*_T_ is higher than 15 dB.

## Results and Discussion

### Design Strategy and Structural Characterizations

**Fig. 1:**
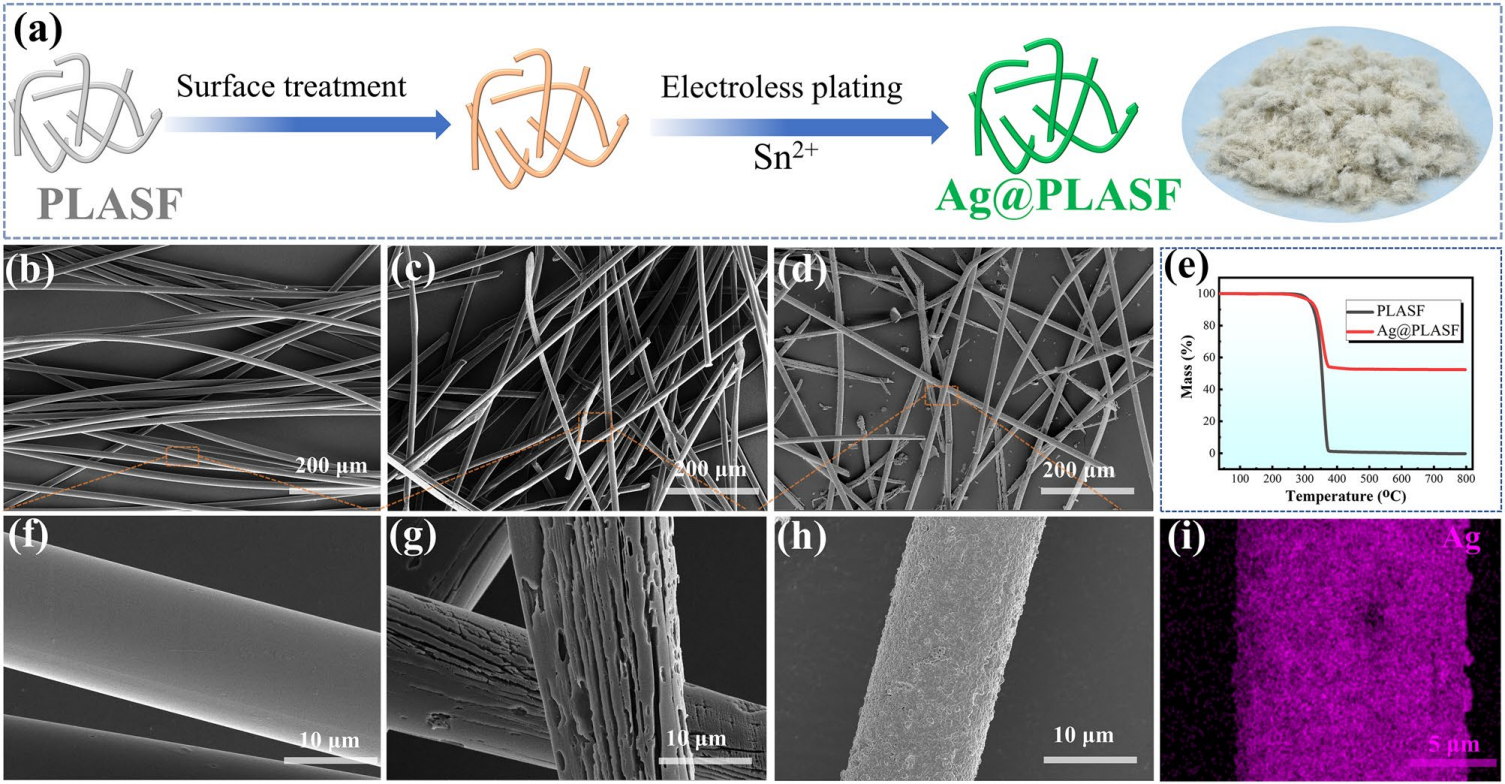
**a** Schematic illustration of the fabrication process for AAF and the digital photograph of AAF; SEM images of **b, f** PLASF, **c, g** treated PLASF, **d, h** AAF; **e** TGA curves of PLASF and AAF; **i** Ag elemental mapping image of AAF

The manufacturing process of AAF is schematically depicted in Fig. 1a, and the details are described in the Experimental Section. The original PLASF with a diameter of about 10 μm exhibits a smooth surface, as shown in Fig. 1b, f. After surface treating process, the external amorphous phases of PLASF are removed and the surface roughness is increased (Fig. 1c, g), which is favorable for Ag nanoparticles plating [40]. Ultimately, the compact Ag nanoparticles are coated on the PLASF, as observed in Fig. 1d and h. The energy-dispersive analysis (EDS) results (Fig. 1i) also demonstrate that the Ag nanoparticles are wrapped uniformly on the individual PLASF. In addition, according to the TGA results (Fig. 1e), the Ag content is about 52.6 wt%. Finally, the synthesized micro-size AAF exhibits yellowish gray color from the exterior surface, as shown on the right side of Fig. 1a. The CNT is well-known as a nano-size conductive filler, which also shows high aspect ratio and the electrical conductivities of AAF and CNT are 6.6 × 10^6^ and 1.5 × 10^6^ S m^−1^ (Fig. S4), demonstrating a great potential in designing the CPCs with ultra-strong EMI protection.

**Fig. 2:**
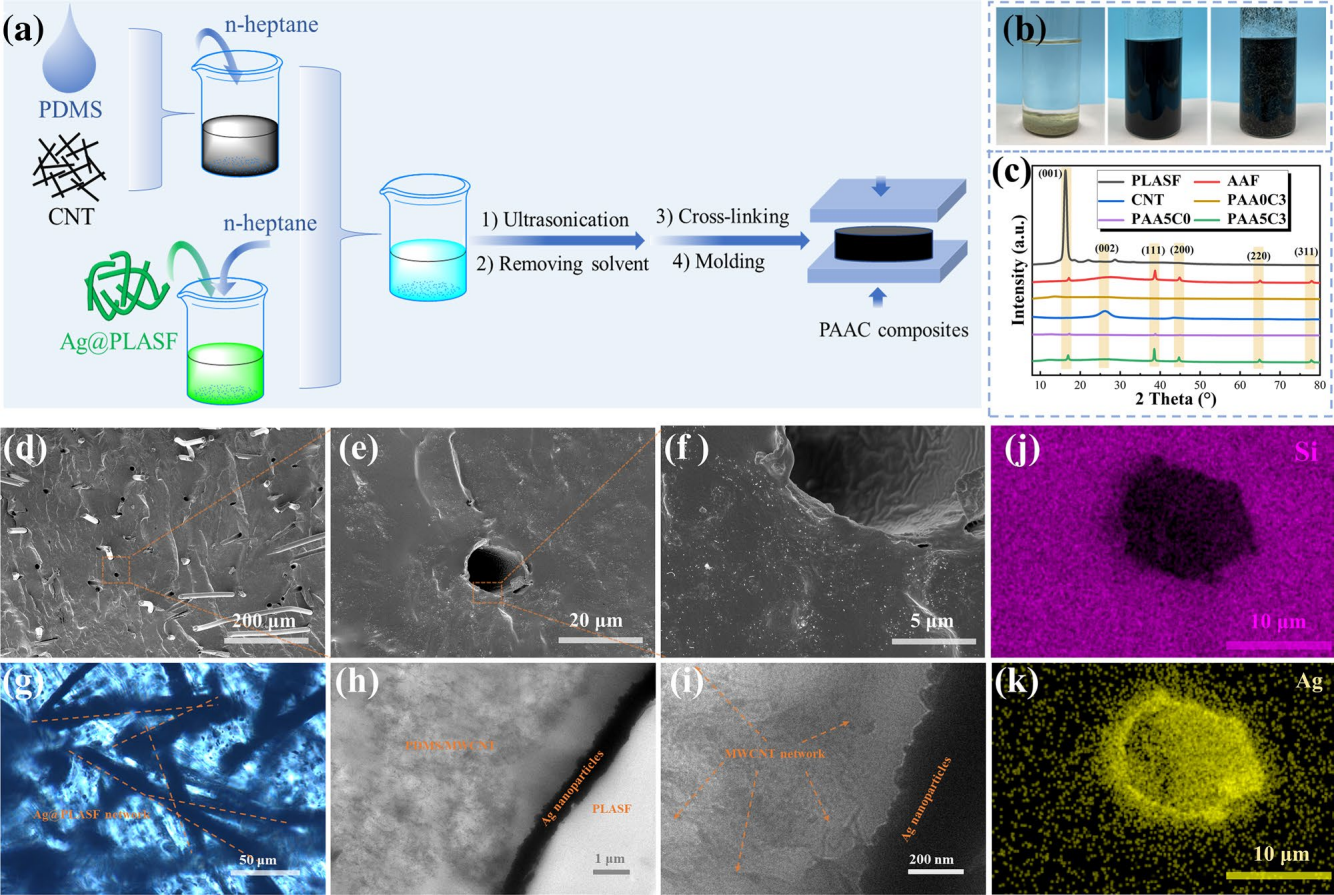
**a** Schematic illustration of the fabrication process of PAAC composites; **b** digital photographs of AAF (left), CNT (middle), AAF/CNT mixture in the n-heptane (right); **c** XRD curves of PLASF, AAF, CNT and the composites containing AAF or CNT; **d-f** SEM images of PAA3C3; **g** the POM image and **h, i** TEM images of PAA3C3 composites with different magnification; **j, k** EDS images of PAA3C3 composites

To mix AAF and CNT well into the PDMS matrix, we propose an efficient process to obtain the ultimate composites, as shown in Fig. 2a. The pronounced process is based on the poor dispersion of AAF and good dispersion of CNT in n-heptane, as presented in the left and middle panel of Fig. 2b. After adding PDMS/CNT dispersion and AAF dispersion in sequence, all fillers are well dispersed in the mixture (the right panel of Fig. 2b) with the aid of sonication. Figure 2c presents the spectra of PAA5C3 composite, showing the superposition of PLASF (001), Ag (111, 200, 311) and CNT (002) spectra, which proves that the above fillers are successfully filled into composites.

Figure 2d–f present the SEM images of the PAA3C3 composite with AAF and CNT, indicating that AAF and CNT are uniformly dispersed in the PDMS matrix, which is also observed in PAAC composites with single-filled AAF or CNT (Fig. S2). It is worth noting from SEM images of the fractured PAAC composites that Ag nanoparticles are tightly embedded in PDMS. Figure 2j-k reveals the EDS results of Fig. 2e, from which Si and Ag elements are detected, demonstrating that the conductive Ag layer in the PDMS matrix is continuous and compact. Further increasing the content of the AAF to 5.0 vol%, the high content of fillers maintains relatively uniform dispersion in the PDMS matrix, as shown in Fig. S1a-c. Figure 2g depicts the POM picture of the PAA3C3 composite. It can be seen that the AAF network is composed of randomly distributed AAF. Figure 2h-i also shows that the dense CNT network consists of abundant CNT, and the ultra-thin Ag layer (400 ~ 500 nm) is in close contact with CNT to construct a multi-scale conductive network.

### Electrical and EMI Shielding Performance of PAAC Composites

**Fig. 3:**
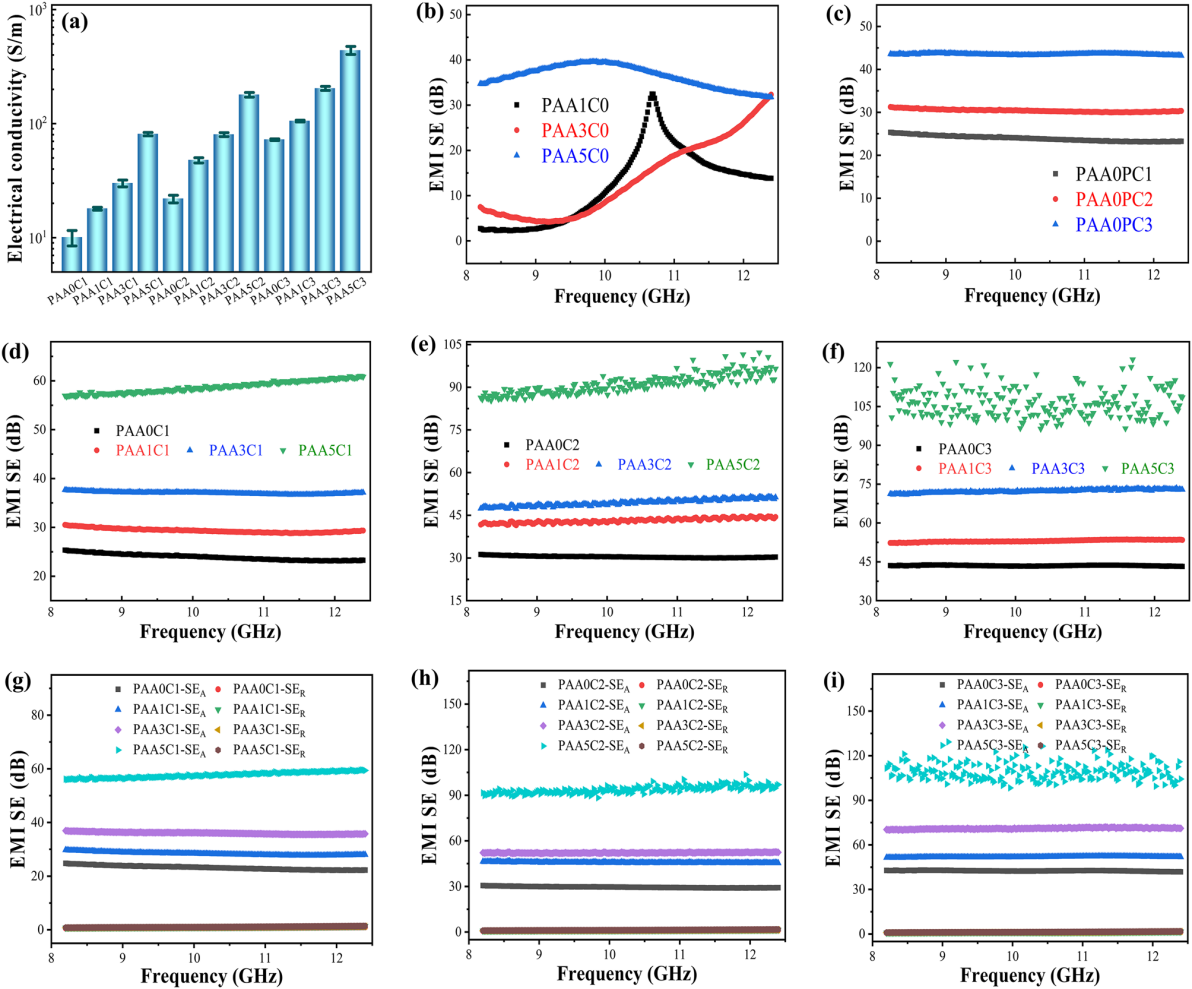
**a** The electrical conductivity of the PAAC composites with different AAF and CNT contents; **b, c** EMI SE of PAAC composites with various single-filled AAF and CNT contents in the X-band; the EMI SE of **d** PAAxC1,** e** PAAxC2, **f** PAAxC3 composites in the X-band frequency range; the absorption loss (*SE*_A_) and reflection loss (*SE*_R_) of **g** PAAxC1, **h** PAAxC2 and **i** PAAxC3 composites in the X-band frequency range

Figure 3a depicts that the electrical conductivity of the composites increases from 10 S m^−1^ for PAA0C1 to 72 S m^−1^ for PAA0C3, with CNT content increasing from 1.0 to 3.0 vol%. The electrical conductivities of the PAA1C0 composite and PAA3C0 composite are 4.5 × 10^–4^ and 0.078 S m^−1^, respectively (Fig. S4). The electrical conductivity of the PAA5C0 composite is 10 S m^−1^ even if the AAF content reached 5.0 vol%, due to the difficulty of utilizing AAF as a micro-size filler to construct conductive networks to transmit electrons. By contrast, the CNT as a nano-size filler can easily design conductive CPCs. Interestingly, the synergistic effect of the multi-scale conductive network between two fillers with different sizes further improves the electrical conductivity of PAAC composites. When the CNT content is 2.0 vol%, the electrical conductivity of the corresponding PAA5C2 composite reaches 179 S m^−1^. As expected, the combination of CNT and AAF can help the acquisition of CPCs with high conductivity. When 5.0 vol% of AAF and 3.0 vol% of CNT are introduced, the electrical conductivity of the resulting PAA5C3 composite reaches 440 S m^−1^.

The EMI shielding ability is usually related to the electrical conductivity of materials. Figure 3b-c display the EMI *SE* in the X-band for PAAC composites filled with AAF or CNT, respectively. The EMI *SE* increases with the increase of conductive filler content. For example, when AAF content increases from 1.0 to 5.0 vol%, the average EMI *SE* of the composite also increases from 12 dB (PAA1C0) to 36 dB (PAA5C0), and when CNT content increases from 1.0 to 3.0 vol%, the average EMI *SE* of composite increases from 24 dB (PAA0C1) to 43 dB (PAA0C3). In the composites with similar filler content and the same thickness of 2.0 mm, the EMI *SE* and electrical conductivity of AAF-filled composites are inferior to those of CNT-filled composites. As shown in Fig. 3d, the EMI *SE* of the PAAC composites is improved to 58 dB with 5.0 vol% of AAF when the CNT content is fixed at 1.0 vol%. When the CNT content further increases to 2.0 vol%, the average EMI *SE* of PAAC composites is enhanced, reaching 91 dB (Fig. 3e). When the AAF and CNT contents are 5.0 and 3.0 vol% respectively, the average EMI SE of the PAA5C3 composite reaches 113 dB. In summary, we have obtained an ultra-strong EMI shielding CPC with a multi-scale conductive network through compounding micro-size AAF and nano-size CNT and filling them into a flexible PDMS matrix.

### Shielding Mechanism and Factors of PAAC Composites

**Fig. 4:**
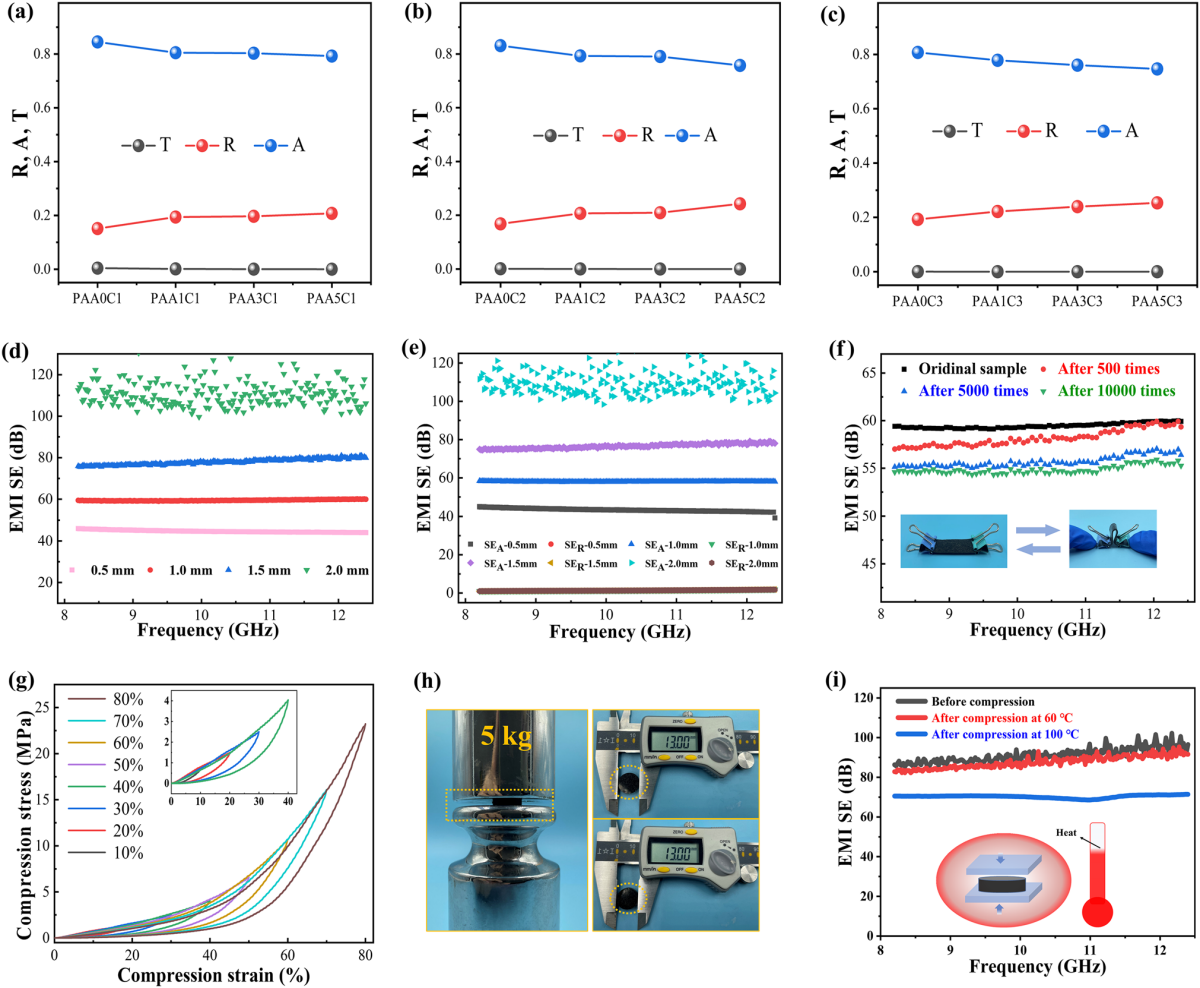
The reflection coefficient (*R*), absorption coefficient (*A*) and transmission coefficient (*T*) at 10.0 GHz for **a** PAAxC1, **b** PAAxC2, **c** PAAxC3 composites; **d** EMI SE, **e** *SE*_A_ and *SE*_R_ of PAA5C3 composite with different thicknesses;** f** EMI SE of PAA5C3 composite before and after bending-releasing cycles with the insets showing a bending-releasing cycle; **g** the compression strain–stress curves of PAA5C2 composite at various strains; **h** Digital photograph illustrating the compression state (left) and the diameter change of the composite (right); **i** EMI SE of PAA5C2 composite before and after compression (load: 5 kg) at 60 and 100 °C

To understand the reason for EMI shielding enhancement of the PAAC composites, the shielding mechanism is studied by evaluating the absorption loss (*SE*_A_) and reflection loss (*SE*_R_). Figure 3g-i presents the *SE*_A_ and *SE*_R_ of PAAC composites with increasing AAF and CNT contents and at the thickness of 2.0 mm. When the CNT content is 1.0 vol%, the *SE*_A_ increases significantly with the increase of AAF content, while the *SE*_R_ increases slightly. For example, the *SE*_A_ increases from 22.3 dB of PAA0C1 composite to 59.4 dB of PAA5C1 composite, while the *SE*_R_ only increases from 1.01 to 1.43 dB, under the frequency of 12.4 GHz (Fig. 3g). As shown in Fig. 3i, when the CNT content further increases to 3.0 vol%, the *SE*_A_ of the PAA5C3 composite reaches 111.1 dB, and *SE*_R_ is only 1.8 dB. As the filler content increases, the *SE*_A_ values enhance greatly, while increases in *SE*_R_ are not obvious, as shown in Fig. S6. In addition, such small *SE*_R_ values indicate a low EM reflection, which can also be verified by the calculation of R-A coefficients. According to the corresponding power coefficient of transmissivity (*T*), reflectivity (*R*) and absorptivity (*A*), the *A* values of PAAC composites are higher than *R* and *T* values, as shown in Fig. 4a-c. Taking the PAA5C3 composite for instance, the A-value is 0.75, which is much larger than 0.25 for the *R*-value at the frequency of 10.0 GHz, demonstrating that a large number of incident EM waves have been absorbed and the microwaves penetrating the PAAC composites have been attenuated by the multi-scale conductive network. These results indicate that absorption loss plays a dominant role in the shielding mechanism of PAAC composites rather than reflection loss.

The thickness of PAAC composites is also an important parameter affecting the EMI shielding performance. As shown in Fig. 4d, with the thickness increasing from 0.5 mm to 2.0 mm, the EMI SE of PAA5C3 composites is boosted significantly from 44.6 to 113 dB. The thickness of PAAC composites has slight influence on the *R, A* and *T* of the shielding mechanism (Fig. S5). The *R* values are ~ 0.25 and the *A* values are 0.75 when the thickness is increased from 0.5 to 2.0 mm. The EMI SE increment is mainly due to the extension of the transmission loss path of EM waves in the PAAC composite, which leads to more EM waves to be absorbed in the multi-scale conductive network (Fig. 4e). For the application of flexible wireless devices, mechanical flexibility and stable EMI shielding performance are the key factors to be considered for EMI shielding composites. Figure 4f portrays the EMI SE of a 1.0-mm PAA5C3 composite tested before and after bending-releasing for 500, 5,000 and 10,000 cycles. The testing results indicate that the PAAC composite remains at 55 dB (90% of the initial value) after 10,000 deformation cycles, suggesting its outstanding flexibility. The compressibility of EMI shielding composites is another important feature for its application in gaskets. Figure 4g shows the typical compression-release curves of PAA5C2 composite with strain from 10 to 80%, which reveals that PAAC composites have good mechanical properties and recovery. The compress stress and modulus of the PAA5C2 composite are enhanced with the increase of compress strains. The compression stress reaches 23.21 MPa at a compression strain of 80% and the modulus reaches as high as 727.4 MPa (Table S2). In addition, the PAA5C2 composite is placed in a hot environment (60 °C) and compressed under a 5 kg-weight (corresponding to the compression strain of 30%) for 7 days, and then the EMI SE of the composite is evaluated. It is discovered that the diameter is unchanged (Fig. 4h), while the EMI SE of the PAA5C2 composite is maintained at 90 dB, as shown in Fig. 4i. When the environmental temperature increases to 100 °C, the EMI SE of the PAA5C2 composite still retains at 71 dB. All the above results indicate that the PAAC composites possess robust mechanical flexibility, good elasticity and ultra-high EMI shielding stability.

**Fig. 5:**
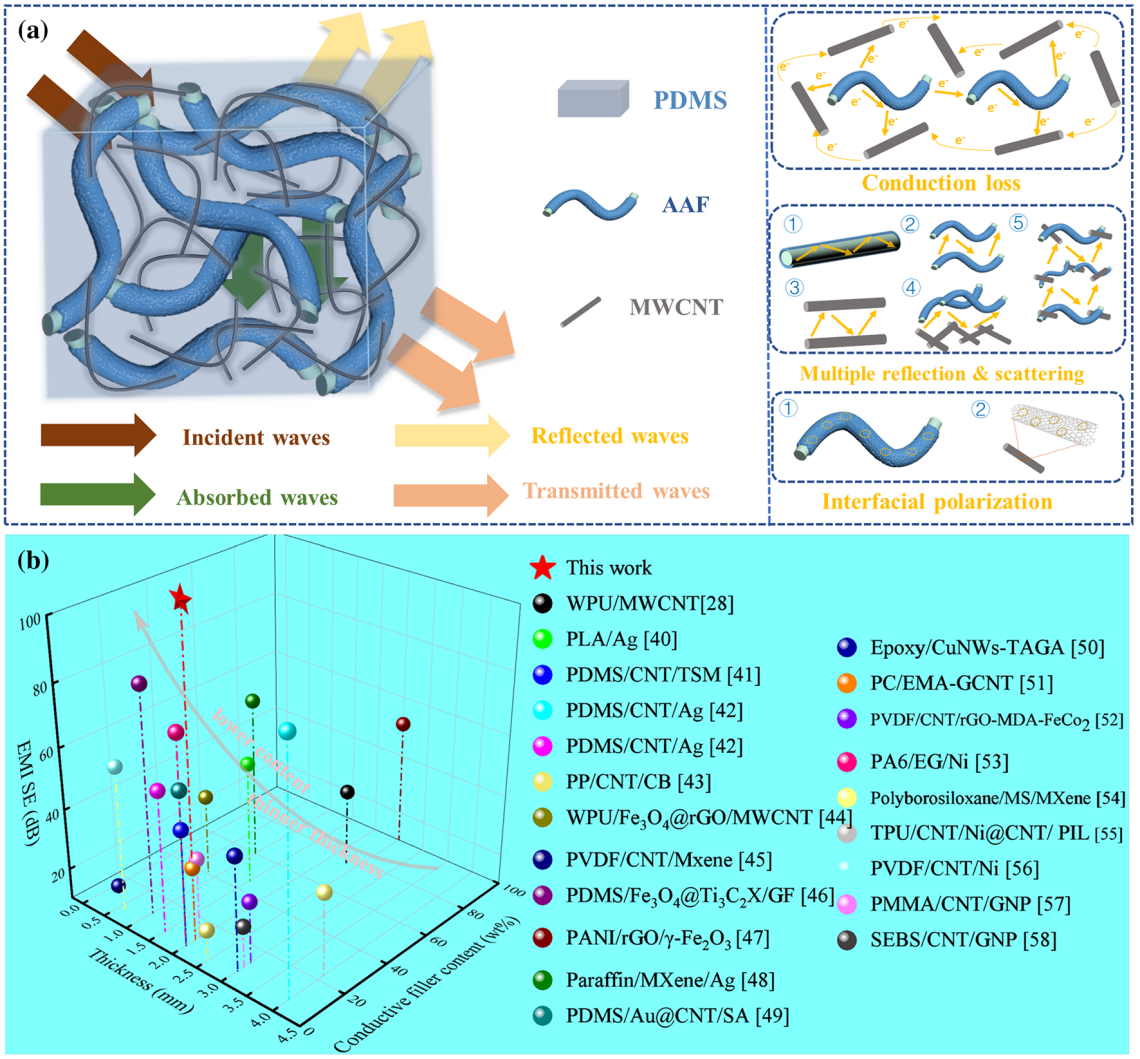
**a** Schematic illustration of transmission loss of EM waves across the PAAC composite with the multi-scale conductive network; **b** Comparison of EMI SE of PAAC composite and previously reported EMI shielding composites with different thicknesses and conductive filler contents. WPU, PLA, TSM, PP, CB, rGO, PVDF, MXene, GF, PANI, SA, CuNWs, TAGA, PC, EMA, GCNT, PA6, EG, MS, TPU, PIL, PMMA and SEBS represent waterborne polyurethane, poly(lactic acid), temperature-sensitive microspheres, polypropylene, carbon black, reduced graphene oxide, poly(vinylidene fluoride), metal carbides/nitrides/carbonitrides, graphene foam, polyaniline, sodium alginate, copper nanowires, thermally annealed graphene aerogel, polycarbonate, ethylene–methyl acrylate, Graphene-MWCNT hybrid filler, polyamide 6, expanded graphite, melamine sponge, thermoplastic polyurethane, polymerizable ionic liquid copolymer, polymethyl methacrylate, poly (styrene-b-ethylene-ran-butylene-b-styrene), respectively

The shielding mechanism is comprehensible in the flexible and compressible PAAC composites with the multi-scale conductive network. Figure 5a shows a schematic diagram of the EM waves transmitted through the PAAC composite. When the incident waves reach the PAAC composite, few EM waves are reflected from the surface, while a large number of EM waves penetrating into the composite. Because the multi-scale conductive network consists of AAF and CNT with high conductivity, the EM waves are absorbed, including conduction loss, multiple reflection and scattering and interfacial effect. The conduction loss includes the charge transfer between the AAF or CNT surfaces, and the electron hopping through the AAF and CNT. The multiple reflection and scattering occur inside the multi-scale conductive network containing AAF and CNT, i.e., the conductive interfaces between the CNT and AAF. The interfacial effect is due to the accumulation of conductivity and space charges of heterogeneous components around the interface between the AAF and CNT. After suffering from these loss situations, few EM waves are transmitted through the PAAC composite, leading to good EMI shielding performance. In short, the PAAC composites containing AAF and CNT show an absorption-dominant shielding mechanism, with high *SE*_A_ and *A* values.

Due to its original multi-scale conductive network, the PAAC composite has unique advantages of ultra-strong EMI shielding property in thickness and conductive filler content. Under the above results, the specific filler contents are calculated and listed in Table S1. For example, the PAA5C3 composite is filled with 4.8 wt% of CNT and 11.9 wt% of AAF, corresponding to an Ag content of 6.3 wt%/0.66 vol%. As can be seen from Fig. 5b and Table S3, previous works have reported the fabrication of outstanding EMI shielding composites with various conductive networks, including high content of CNT or Ag nanoparticles, CNT and Ag nanoparticles, as well as CNT and other conductive fillers. For example, Zhang et al. have reported a type of PDMS/CNT/Ag composites based on plating Ag on CNTs. The EMI SE of the composites reaches as high as 90 dB, but both the total filler content and the thickness are quite high, which are 17.0 wt% and up to 4.0 mm, respectively. It is worth noting that PAAC composites filled with micro-size AAF and nano-size CNT show immense potential for designing outstanding EMI shielding composites. Consequently, the flexible PAAC composites with the multi-scale conductive network not only exhibit stable ultra-strong EMI shielding performance but also have excellent potential in flexible wireless devices.

## Conclusions

In this work, we have constructed a multi-scale conductive network and prepared PAAC composites with different contents of micro-size AAF and nano-size CNT by solution blending and compression molding. The AAF microstructure reveals that the silver nanoparticles are successfully plated on the PLASF through the etching method. AAF and CNT are uniformly dispersed in the flexible PDMS matrix to construct multi-scale conductive networks. As a result, under the appropriate thickness of 2.0 mm and low filler content of 11.1 wt%, the PAAC composites show outstanding electrical conductivity of 440 S m^−1^ and superior EMI shielding performance of up to 113 dB. The EMI SE enhancement is mainly owing to the increase in conduction loss, multiple reflection loss and interfacial polarization inside the PAAC composites. As expected, even with 7 days of compression in a simulated aging environment, or bending-releasing 10,000 cycles of deformation, the PAAC composites still maintain (> 90%) good EMI shielding performance because of their admirable compressibility and flexibility. This strategy will provide important guidance for designing flexible and superior EMI shielding CPCs with multi-scale conductive networks to protect the workspace, environment and sensitive circuits against radiation for flexible electronic devices.

### Supplementary Information

Below is the link to the electronic supplementary material.

Supplementary file1 (PDF 617 kb)
