# Supplementary material for: Flexible Polydimethylsiloxane Composite with Multi-Scale Conductive Network for Ultra-Strong Electromagnetic Interference Protection
Source: Nanomicro Lett. 2022 Dec 29;15:15. doi: 10.1007/s40820-022-00990-7 (PMC9800674; doi:10.1007/s40820-022-00990-7)
Supplement: Supplementary file 1 — Supplementary file1 (PDF 617 kb) [file 40820_2022_990_MOESM1_ESM.pdf]

Supporting Information for

# Flexible Polydimethylsiloxane Composite with Multi-Scale Conductive Network for Ultra-Strong Electromagnetic Interference Protection

Jie Li[1], He Sun[1], Shuang-Qin Yi[1], Kang-Kang Zou[2], Dan Zhang[1], Gan-Ji Zhong[1], Ding-Xiang Yan[2,*], and Zhong-Ming Li[1,*]

[1] College of Polymer Science and Engineering, State Key Laboratory of Polymer Materials Engineering, Sichuan University, Chengdu 610065, P. R. China

[2] School of Aeronautics and Astronautics, Sichuan University, Chengdu 610065, P. R. China

*Corresponding authors. E-mail: yandingxiang@scu.edu.cn (D. X. Yan), zmli@scu.edu.cn (Z. M. Li)

## Supplementary Tables and Figures

**Table S1** Calculation of the filler contents for PAAC composites

| Sample | CNT (vol%) | AAF (vol%) | Ag (vol%) | AAF (wt%) | Ag (wt%) | CNT (wt%) |
|---|---|---|---|---|---|---|
| PAA0C1 | 1.0 | 0 | 0 | 0 | 0 | 1.7 |
| PAA0C2 | 2.0 | 0 | 0 | 0 | 0 | 3.4 |
| PAA0C3 | 3.0 | 0 | 0 | 0 | 0 | 5.1 |
| PAA1C0 | 0 | 1.0 | 0.13 | 2.6 | 1.4 | 0 |
| PAA3C0 | 0 | 3.0 | 0.39 | 7.5 | 4.0 | 0 |
| PAA5C0 | 0 | 5.0 | 0.66 | 12.2 | 6.4 | 0 |
| PAA1C1 | 1.0 | 1.0 | 0.13 | 2.6 | 1.3 | 1.7 |
| PAA3C1 | 1.0 | 3.0 | 0.39 | 7.5 | 3.9 | 1.7 |
| PAA5C1 | 1.0 | 5.0 | 0.66 | 12.1 | 6.4 | 1.6 |
| PAA1C2 | 2.0 | 1.0 | 0.13 | 2.5 | 1.3 | 3.4 |
| PAA3C2 | 2.0 | 3.0 | 0.39 | 7.4 | 3.9 | 3.3 |
| PAA5C2 | 2.0 | 5.0 | 0.66 | 12.0 | 6.3 | 3.2 |
| PAA1C3 | 3.0 | 1.0 | 0.13 | 2.5 | 1.3 | 5.1 |
| PAA3C3 | 3.0 | 3.0 | 0.39 | 7.4 | 3.9 | 4.9 |
| PAA5C3 | 3.0 | 5.0 | 0.66 | 11.9 | 6.3 | 4.8 |

According to the calculated mass fraction of Ag, the AAF, Ag and CNT contents were respectively calculated based on Eqs. S1-S6 as follows:

$$v_{CNT} = \frac{W_{CNT}/\rho_{CNT}}{W_{AAF}\times\frac{w_{Ag1}}{\rho_{Ag}}+W_{AAF}\times\frac{(1-w_{Ag1})}{\rho_{PLASF}}+\frac{W_{CNT}}{\rho_{CNT}}+\frac{W_{PDMS}}{\rho_{PDMS}}} \times 100\% \quad \text{(S1)}$$

$$v_{AAF} = \frac{W_{AAF}\times\frac{w_{Ag1}}{\rho_{Ag}}+W_{AAF}\times\frac{(1-w_{Ag1})}{\rho_{PLASF}}}{W_{AAF}\times\frac{w_{Ag1}}{\rho_{Ag}}+W_{AAF}\times\frac{(1-w_{Ag1})}{\rho_{PLASF}}+\frac{W_{CNT}}{\rho_{CNT}}+\frac{W_{PDMS}}{\rho_{PDMS}}} \times 100\% \quad \text{(S2)}$$

$$v_{Ag} = \frac{W_{AAF}\times w_{Ag1}/\rho_{Ag}}{W_{AAF}*\frac{w_{Ag1}}{\rho_{Ag}}+W_{AAF}\times\frac{(1-w_{Ag1})}{\rho_{PLASF}}+\frac{W_{CNT}}{\rho_{CNT}}+\frac{W_{PDMS}}{\rho_{PDMS}}} \times 100\% \quad \text{(S3)}$$

$$w_{Ag2} = \frac{W_{AAF}\times w_{Ag1}}{W_{AAF}+W_{CNT}+W_{PDMS}} \times 100\% \quad \text{(S4)}$$

$$w_{AAF} = \frac{W_{AAF}}{W_{AAF}+W_{CNT}+W_{PDMS}} \times 100\% \quad \text{(S5)}$$

$$w_{CNT} = \frac{W_{AAF}}{W_{AAF}+W_{CNT}+W_{PDMS}} \times 100\% \quad \text{(S6)}$$

Where $W_{AAF}$, $W_{CNT}$, $W_{PDMS}$ were the calculated weight of AAF, CNT and PDMS; $\rho_{Ag}$, $\rho_{PLASF}$, $\rho_{CNT}$, $\rho_{PDMS}$ were the density of Ag, PLASF, CNT and PDMS; $w_{Ag1}$, $w_{Ag2}$, $w_{AAF}$, $w_{CNT}$ were the mass fraction of Ag in the AAF, and Ag, AAF, CNT in the PAAC composites; $v_{Ag}$, $v_{AAF}$, $v_{CNT}$ were the volume content of Ag in the AAF, and Ag, AAF, CNT in the PAAC composites; respectively. The densities of Ag, PLASF and CNT are 10.49, 1.24 and 1.75 g/cm$^3$.

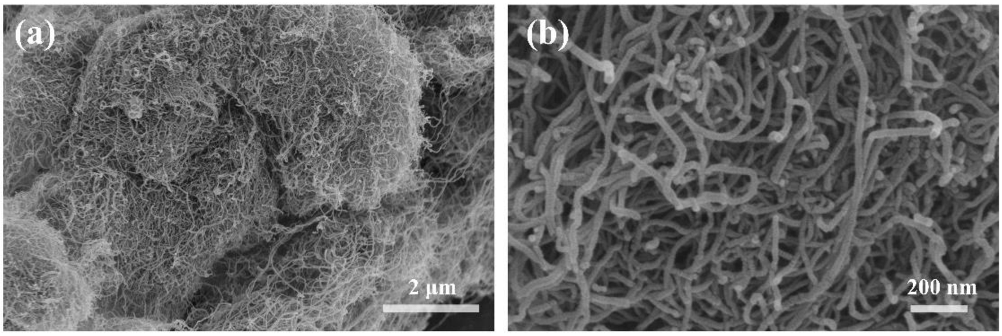


**Fig. S1 a, b** SEM images of CNT with different magnifications

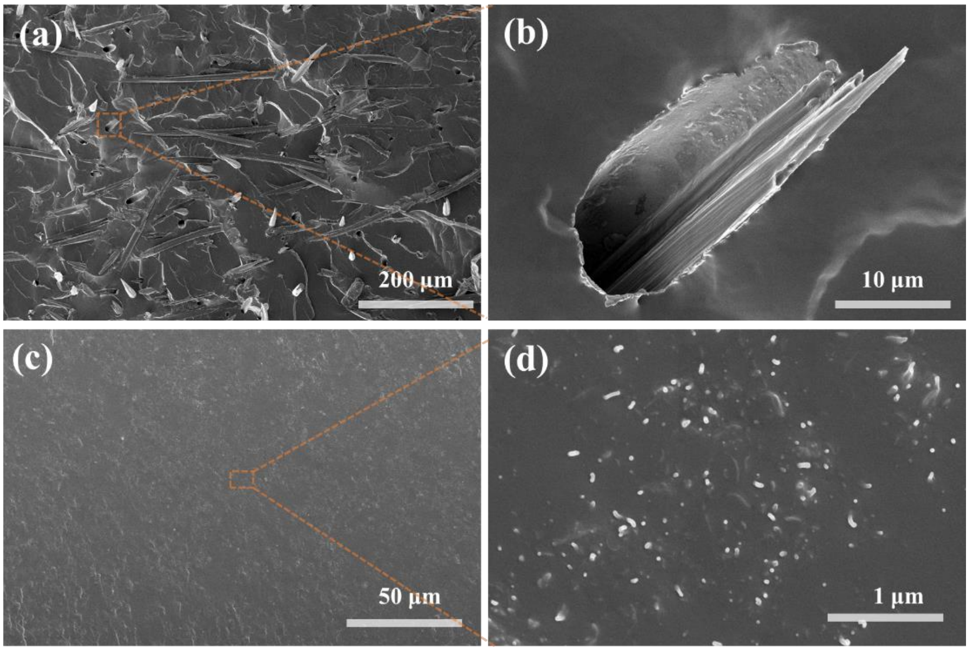


**Fig. S2** SEM images of **a, b** PAA5C0 and **c, d** PAA0C3 with different magnifications

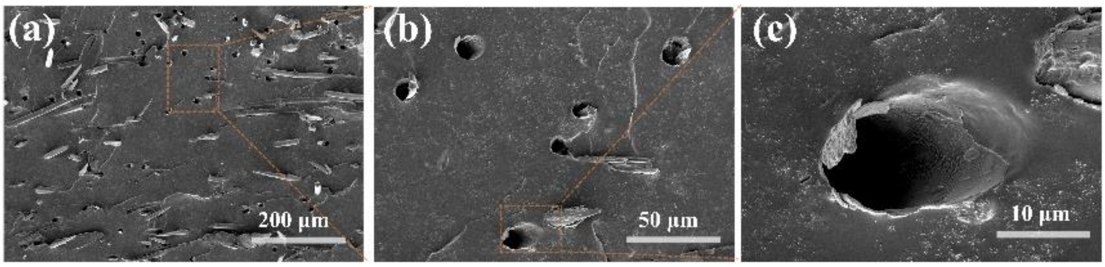


**Fig. S3 a-c** SEM images of PAA5C3 composites with different magnifications.

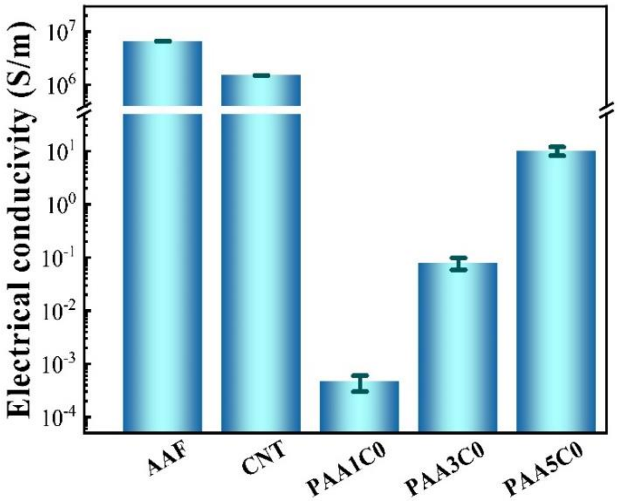


**Fig. S4** The electrical conductivity of AAF, CNT and PAAxC0 composites

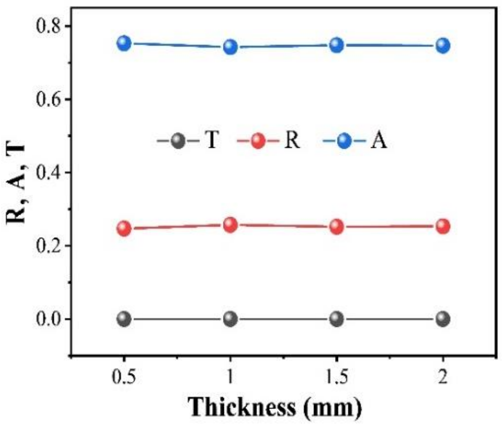


**Fig. S5** The reflection coefficient (R), absorption coefficient (A) and transmission coefficient (T) at 10.0 GHz of PAA5C3 composites at different thicknesses

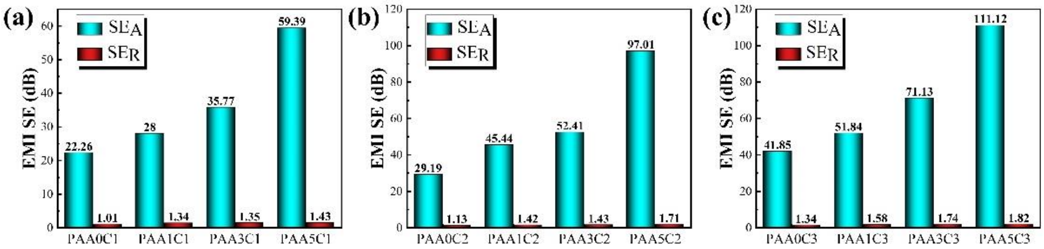


**Fig. S6** The absorption loss ($SE_A$) and reflection loss ($SE_R$) of **a** PAAxC1, **b** PAAxC2, **c** PAAxC3 composites at the frequency of 10.0 GHz

**Table S2** The compression stress and modulus of PAA5C2 composite at different strains

| Compression strain (%) | Compression stress (MPa) | Compression modulus (MPa) |
|---|---|---|
| 10 | 0.68 | 66.2 |
| 20 | 1.49 | 77.7 |
| 30 | 2.50 | 89.1 |
| 40 | 4.04 | 139.9 |
| 50 | 6.56 | 230.0 |
| 60 | 10.68 | 386.0 |
| 70 | 16.08 | 543.5 |
| 80 | 23.21 | 727.4 |

**Table S3** Comparison of other EMI shielding composites in recent 5 years

| Sample | Content (wt%) | Thickness (mm) | EMI SE (dB) | Frequency (GHz) | Refs. |
|---|---|---|---|---|---|
| PDMS/CNT/Ag | 11.1 | 2 | 113 | 8.2-12.4 | This work |
| PDMS/CNT/Ag | 9.5 | 2 | 82 | 8.2-12.4 | |
| PDMS/CNT | 5.1 | 2 | 43 | 8.2-12.4 | |
| PDMS/Ag | 6.4 | 2 | 36 | 8.2-12.4 | |
| PDMS/CNT/GNP | 20 | 3.6 | 66 | 8.2-12.4 | [S1] |
| PDMS/CNT/Carbon aerogel | 2 | 2 | 20 | 8.2-12.4 | [S2] |
| PDMS/Au@CNT/SA | 6 | 2 | 60 | 8.0-12.0 | [S3] |
| PDMS/CNT/Ag | 17 | 4 | 90 | 8.2-12.4 | [S4] |
| PDMS/CNT/Ag | 17 | 1.5 | 56 | 8.2-12.4 | [S4] |
| PDMS/CNT/TSM | 6 | 2 | 47.8 | 8.2-12.4 | [S5] |
| PDMS/Graphene | 1.5 | 4.8 | 45 | 8.2-12.4 | [S6] |
| PDMS/HOGF | 21.46 | 5 | 96 | 8.2-12.4 | [S7] |
| PDMS/$Fe_3O_4$-$Ti_3C_2T_X$/GF | 11.53 | 1 | 83.6 | 8.2-12.4 | [S8] |
| SEBS/CNT/GNP | 10 | 3 | 23.3 | 8.2-12.4 | [S9] |
| SEBS/CNT/GNP | 90 | 2 | 25 | 8.2-12.4 | [S10] |
| WPU/CNT | 76.2 | 2.3 | 35 | 8.2-12.4 | [S11] |
| PLA/Ag | 34.44 | 1.5 | 50 | 8.2-12.4 | [S12] |
| PP/CNT/CB | 5 | 2.5 | 20 | 8.2-12.4 | [S13] |
| WPU/$Fe_3O_4$@rGO/CNT | 41.2 | 0.8 | 35.9 | 8.2-12.4 | [S14] |
| PVDF/CNT/MXene | 12 | 0.2 | 12.6 | 18-26 | [S15] |
| PANI/rGO/$\gamma$-$Fe_2O_3$ | 100 | 2.5 | 51 | 8.2-12.4 | [S16] |
| Paraffin/MXene/Ag | 60 | 1 | 62.7 | 8.2-18 | [S17] |
| epoxy/CuNWs-TAGA | 7.2 | 3 | 47 | 8.2-12.4 | [S18] |
| PC/EMA-GCNT | 10 | 2 | 34 | 8.2-12.4 | [S19] |
| PVDF/CNT/rGO- $FeCo_2$ | 13 | 3 | 30 | 8.2-18 | [S20] |
| PA6/EG/Ni | 5.9 | 2 | 77.3 | 8.2-12.4 | [S21] |
| Polyborosiloxane/MS/MXene | 22.75 | 4 | 37 | 8.2-12.4 | [S22] |
| TPU/CNTs/Ni@CNT | 20 | 2 | 69.9 | 8.2-12.4 | [S23] |
| PVDF/CNT/Ni | 7 | 0.6 | 57.3 | 18-26 | [S24] |
| PMMA/MWCNT/GNP | 12 | 2 | 36 | 8.0-12.0 | [S25] |
| Ecoflex/LM | 67.2 | 2 | 81.6 | 8.2-12.4 | [S26] |

* GNP, SA, TSM, HOGF, GF, SEBS, WPU, PLA, PP, CB, rGO, PVDF, MXene, ]PANI, CuNWs, TAGA, PC, EMA, GCNT, PA6, EG, MS, TPU, PMMA and LM represent graphene nanoplates, sodium alginate, temperature-sensitive microspheres, highly oriented graphite frameworks, graphene foam, poly (styrene-b-ethylene-ran-butylene-b-styrene), waterborne polyurethane, poly(lactic acid), polypropylene, carbon black, reduced graphene oxide, poly(vinylidene fluoride), metal carbides/nitrides/ carbonitrides, polyaniline, copper nanowires, thermally annealed graphene aerogel, polycarbonate, ethylene-methyl acrylate, Graphene-MWCNT hybrid filler, polyamide 6, expanded graphite, melamine sponge, thermoplastic polyurethane, polymerizable ionic liquid copolymer, polymethyl methacrylate and liquid metal, respectively.

## Supplementary References

[S1] Q. Wei, S. Pei, X. Qian, H. Liu, Z. Liu et al., Superhigh electromagnetic interference shielding of ultrathin aligned pristine graphene nanosheets film. Adv. Mater. **32**(14), e1907411 (2020). https://doi.org/10.1002/adma.201907411

[S2] M. Chen, L. Zhang, S. Duan, S. Jing, H. Jiang et al., Highly conductive and flexible polymer composites with improved mechanical and electromagnetic interference shielding performances. Nanoscale **6**(7), 3796-3803 (2014). https://doi.org/10.1039/c3nr06092f

[S3] X. Lei, X. R. Zhang, A. R. Song, S. Gong, Y. Wang et al., Investigation of electrical conductivity and electromagnetic interference shielding performance of Au@CNT/sodium alginate/polydimethylsiloxane flexible composite. Composites, Part A **130**, 105762 (2020). https://doi.org/10.1016/j.compositesa.2019.105762

[S4] J. P. Zhang, H. T. Li, T. Xu, J. J. Wu, S. L. Zhou et al., Homogeneous silver nanoparticles decorating 3D carbon nanotube sponges as flexible high-performance electromagnetic shielding composite materials. Carbon **165**, 404-411 (2020). https://doi.org/10.1016/j.carbon.2020.04.043

[S5] J. H. Cai, X. H. Tang, X. D. Chen, M. Wang, Temperature and strain-induced tunable electromagnetic interference shielding in polydimethylsiloxane/multi-walled carbon nanotube composites with temperature-sensitive microspheres. Composites, Part A. **140**, 106188 (2021). https://doi.org/10.1016/j.compositesa.2020.106188

[S6] Z. Wang, W. Yang, R. Liu, X. Zhang, H. Nie et al., Highly stretchable graphene/polydimethylsiloxane composite lattices with tailored structure for strain-tolerant EMI shielding performance. Compos. Sci. Technol. **206**, 108652 (2021). https://doi.org/10.1016/j.compscitech.2021.108652

[S7] F. Zhang, C. Li, Y. Zhang, Y. Sun, X. Yao et al., Facile preparation of large-scale expanded graphite/polydimethylsiloxane composites for Highly-efficient electromagnetic interference shielding. J Mater. Chem. A (2022). https://doi.org/10.1039/d2ta06263a

[S8] V. T. Nguyen, B. K. Min, Y. Yi, S. J. Kim, C. G. Choi, MXene($Ti_3C_2T_X$)/graphene/PDMS composites for multifunctional broadband electromagnetic interference shielding skins. Chem. Eng. J. **393**, 124608 (2020). https://doi.org/10.1016/j.cej.2020.124608

[S9] S. Kuester, N. R. Demarquette, J. C. Ferreira, B. G. Soares, G. M. O. Barra, Hybrid nanocomposites of thermoplastic elastomer and carbon nanoadditives for electromagnetic shielding. Eur. Polym. J. **88**, 328-339 (2017). https://doi.org/10.1016/j.eurpolymj.2017.01.023

[S10] Y. Xu, S. Tang, J. Pan, J. Bao, A. Zhang, Reversibly cross-linked SEBS/carbon hybrid composite with excellent solvent-proof and electromagnetic shielding properties. Mater. Des. **146**(1-11 (2018). https://doi.org/10.1016/j.matdes.2018.02.071

[S11] Z. Zeng, H. Jin, M. Chen, W. Li, L. Zhou et al., Lightweight and anisotropic porous mwcnt/wpu composites for ultrahigh performance electromagnetic interference shielding. Adv. Funct. Mater. **26**(2), 303-310 (2016). https://doi.org/10.1002/adfm.201503579

[S12] K. Zhang, H.-O. Yu, K.-X. Yu, Y. Gao, M. Wang et al., A facile approach to constructing efficiently segregated conductive networks in poly(lactic acid)/silver nanocomposites via silver plating on microfibers for electromagnetic interference shielding. Compos. Sci. Technol. **156**, 136-143 (2018). https://doi.org/10.1016/j.compscitech.2017.12.037

[S13] J. J. Ju, T. R. Kuang, X. P. Ke, M. Zeng, Z. Chen et al., Lightweight multifunctional polypropylene/carbon nanotubes/carbon black nanocomposite foams with segregated structure, ultralow percolation threshold and enhanced electromagnetic interference shielding performance. Compos. Sci. Technol. **193**, 108116 (2020). https://doi.org/10.1016/j.compscitech.2020.108116

[S14] A. Sheng, W. Ren, Y. Yang, D.-X. Yan, H. Duan et al., Multilayer WPU conductive composites with controllable electro-magnetic gradient for absorption-dominated electromagnetic interference shielding. Composites, Part A. **129**, 105692 (2020). https://doi.org/10.1016/j.compositesa.2019.105692

[S15] R. S. Li, L. Ding, Q. Gao, H. M. Zhang, D. Zeng et al., Tuning of anisotropic electrical conductivity and enhancement of EMI shielding of polymer composite foam via $CO_2$-assisted delamination and orientation of MXene. Chem. Eng. J. **415**, 128930 (2021). https://doi.org/10.1016/j.cej.2021.128930

[S16] A. P. Singh, M. Mishra, P. Sambyal, B. K. Gupta, B. P. Singh et al., Encapsulation of $\gamma$-$Fe_2O_3$ decorated reduced graphene oxide in polyaniline core–shell tubes as an exceptional tracker for electromagnetic environmental pollution. J Mater. Chem. A **2**(10), 3581-3593 (2014). https://doi.org/10.1039/c3ta14212d

[S17] K. Rajavel, Y. G. Hu, P. L. Zhu, R. Sun, C. P. Wong, MXene/metal oxides-Ag ternary nanostructures for electromagnetic interference shielding. Chem. Eng. J. **399**, 125791 (2020). https://doi.org/10.1016/j.cej.2020.125791

[S18] X. T. Yang, S. G. Fan, Y. Li, Y. Q. Guo, Y. G. Li et al., Synchronously improved electromagnetic interference shielding and thermal conductivity for epoxy nanocomposites by constructing 3D copper nanowires/thermally annealed graphene aerogel framework. Composites, Part A **128**, 105670 (2020). https://doi.org/10.1016/j.compositesa.2019.105670

[S19] N. Bagotia, V. Choudhary, D. K. Sharma, Synergistic effect of graphene/multiwalled carbon nanotube hybrid fillers on mechanical, electrical and EMI shielding properties of polycarbonate/ethylene methyl acrylate nanocomposites. Composites, Part B **159**, 378-388 (2019). https://doi.org/10.1016/j.compositesb.2018.10.009

[S20] I. Arief, S. Biswas, S. Bose, FeCo-anchored reduced graphene oxide framework-based soft composites containing carbon nanotubes as highly efficient microwave absorbers with excellent heat dissipation ability. ACS Appl. Mater. Interfaces **9**(22), 19202-19214 (2017). https://doi.org/10.1021/acsami.7b04053

[S21] H. J. Duan, P. Y. He, H. X. Zhu, Y. Q. Yang, G. Z. Zhao et al., Constructing 3D carbon-metal hybrid conductive network in polymer for ultra-efficient electromagnetic interference shielding. Composites, Part B **212**(108690 (2021). https://doi.org/10.1016/j.compositesb.2021.108690

[S22] M. Sang, Y. X. Wu, S. Liu, L. F. Bai, S. Wang et al., Flexible and lightweight melamine sponge/MXene/polyborosiloxane (MSMP) hybrid structure for high-performance electromagnetic interference shielding and anti-impact safe-guarding. Composites, Part B **211**, 108669 (2021). https://doi.org/10.1016/j.compositesb.2021.108669

[S23] G. Sang, P. Xu, T. Yan, V. Murugadoss, N. Naik et al., Interface Engineered Microcellular Magnetic Conductive Polyurethane Nanocomposite Foams for Electromagnetic Interference Shielding. Nano-Micro Lett. **13**(1), 153 (2021). https://doi.org/10.1007/s40820-021-00677-5

[S24] B. Zhao, S. Wang, C. Zhao, R. Li, S. M. Hamidinejad et al., Synergism between carbon materials and Ni chains in flexible poly(vinylidene fluoride) composite films with high heat dissipation to improve electromagnetic shielding properties. Carbon **127**, 469-478 (2018). https://doi.org/10.1016/j.carbon.2017.11.032

[S25] H. Zhang, G. Zhang, M. Tang, L. Zhou, J. Li et al., Synergistic effect of carbon nanotube and graphene nanoplates on the mechanical, electrical and electromagnetic interference shielding properties of polymer composites and polymer composite foams. Chem. Eng. J. **353**, 381-393 (2018). https://doi.org/10.1016/j.cej.2018.07.144

[S26] B. Yao, W. Hong, T. Chen, Z. Han, X. Xu et al., Highly Stretchable Polymer Composite with Strain-Enhanced Electromagnetic Interference Shielding Effectiveness. Adv. Mater. **32**(14), e1907499 (2020). https://doi.org/10.1002/adma.201907499
